# Sarcoidosis with prevalent and severe joint localization: a case report

**DOI:** 10.1186/s40248-016-0064-1

**Published:** 2016-06-29

**Authors:** Livio G. Moccia, Sabrina Castaldo, Emanuela Sirignano, Maddalena Napolitano, Enrica Barra, Alessandro Sanduzzi

**Affiliations:** Division of Pneumology, Department of Clinical Medicine and Surgery, University “Federico II” Medical School, Naples, Italy; Division of Dermatology, Department of Clinical Medicine and Surgery, University “Federico II” Medical School, Naples, Italy; Department of Pathology, High Speciality Hospital “V. Monaldi”, Naples, Italy

**Keywords:** Bronchoalveolar lavage, Corticosteroid therapy, High resolution chest tomography, Mediastinal lymph nodes, Metacarpophalangeal joints, Sarcoidosis-Systemic granulomatous disease

## Abstract

**Background:**

Sarcoidosis is a systemic granulomatous disease of unknown origin, characterized by the formation of granulomas without central necrosis. Each organ and tissue can be affected by the disease, but in most cases mainly the lungs and mediastinal lymph nodes but also skin, heart, eyes and joints are involved, the latter are mainly the metacarpophalangeal joints and bone lesions are often associated with involvement of the overlying skin. The diagnosis is often of exclusion, based on clinical and radiological suspicion, and should be confirmed by biopsy, although in each case it is necessary to exclude other possible causes of granulomatosis, including infections by mycobacteria. Here it is reported a case of particularly aggressive sarcoidosis with primitive involvement of the small joints of the hands and feet, and mediastinal lymph nodes.

**Case presentation:**

The subject, a man, 60 years old, born in Morocco but living in Italy for many years, presented important involvement of bone structures and soft periarticular tissue, and was affected by the formation of granulomas without “caseum necrosis”. The painful symptoms and the skin ulceration had led to surgical amputation of the distal phalanges of most fingers of his hands and feet, but with subsequent resurgence of lesions in acral locations after surgery. The PET/CT scan showed an amount of radiotracer in mediastinal lymph nodes, while the lymph nodes sampled by TBNA were normal and the CD4/CD8 ratio was less than 3 in the bronchoalveolar lavage. We ruled out any possible infectious cause, including mycobacterial infection (both tubercular and atypical), so the patient was treated with systemic corticosteroids, with an excellent clinical and radiological response.

**Conclusions:**

Such a case shows how the disease can have variable expressions, without primitive lung involvement; therefore, it should be necessary to consider any possible, unpredictable localization of the disease.

## Background

Sarcoidosis is a multisystem disease of unknown origin, characterized by the formation of immune granulomas without central necrosis with primary involvement of the lungs and mediastinal lymph nodes[1, 2, 3]. Osteo-articular manifestations are uncommon, and are often associated with other symptoms [4, 5]. This involvement in patients with sarcoidosis is around 3–13 %, and is clinically relevant only in 2–5 % of cases, presenting frequently an asymptomatic course. The osteo-articular manifestations are often mild and highly variable: despite this, these forms take advantage of corticosteroid therapy, with a complete remission of the disease and a clear improvement of symptoms. In this case, on the contrary, the patient underwent several surgical amputation of fingers of hands and toes affected by the disease, with subsequent resurgence of lesions in acral locations after surgery. Only the correct diagnosis of sarcoidosis and the use of prolonged corticosteroid therapy resulted in a significant clinical and radiological improvement.

## Case presentation

**Fig. 1:**
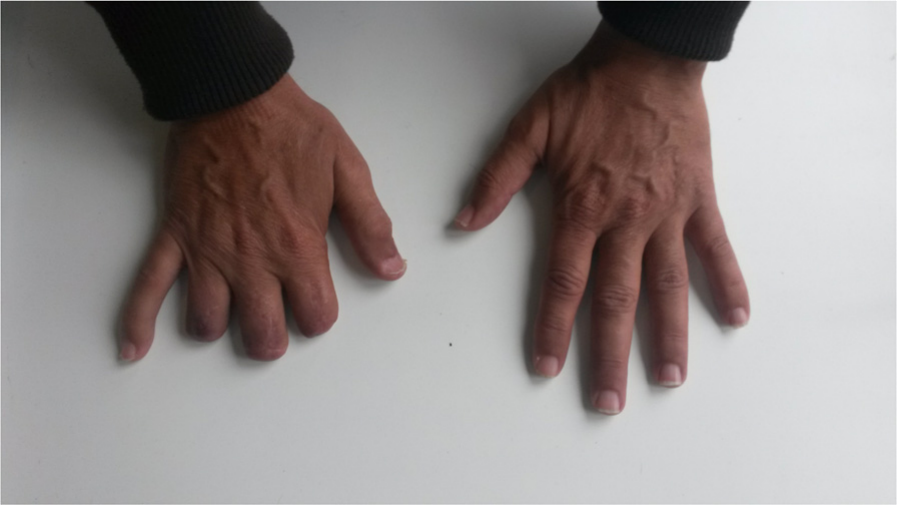
Between 2008 and 2012 the patient underwent several surgical amputations of fingers: distal phalanges of second, third and fourth fingers of right hand

**Fig. 2:**
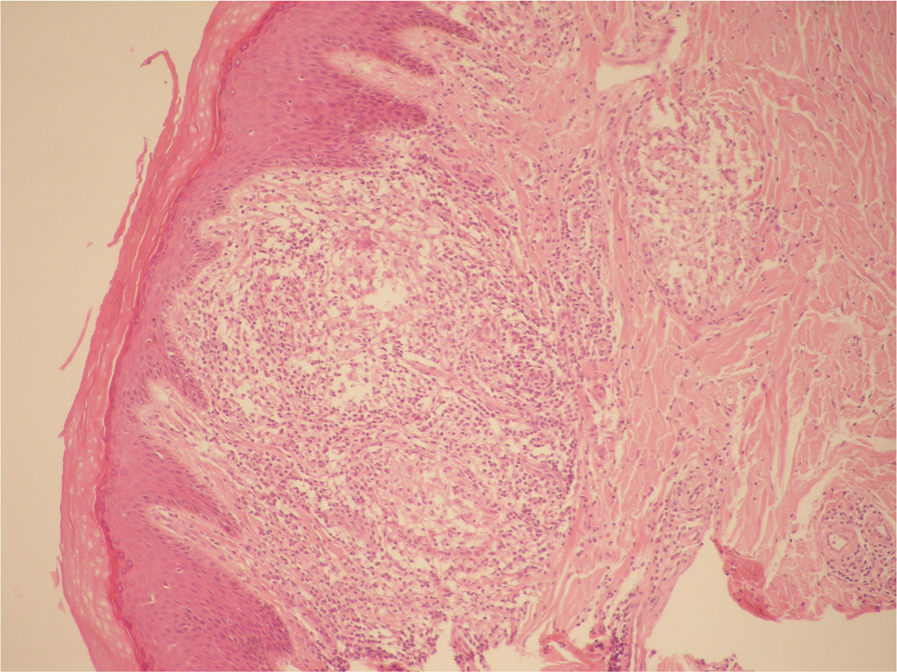
Skin's section: a chronic granulomatous process, with epitelioid histiocytes, rare multinuclear giant cells sorrounded by lymphocytes without central necrosis

**Fig. 3:**
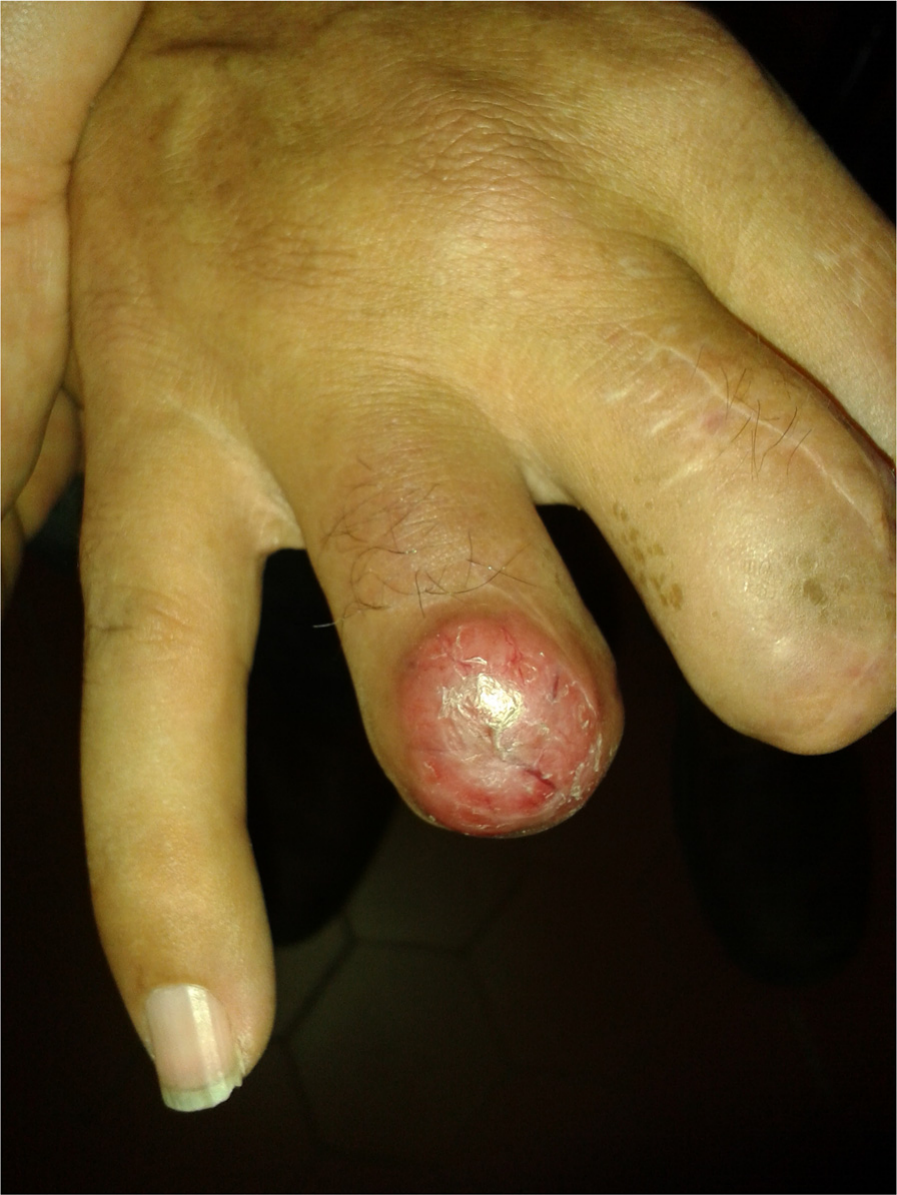
The lesions tended to recur at proximal metacarpophalangeal joints of fingers amputated of distal parts

Herein, we describe the case of a man, 60 years old, never smoker, peddler, born in Morocco but resident in Italy for almost 30 years with sporadic return to his country, with worsening dyspnea since 2008, diffuse arthralgias and nodules tending to ulceration at distal phalangeal joints of his hands and feet. Due to pain and severe functional limitation, at this site, between 2008 and 2012 the patient underwent several surgical amputations of fingers: the distal phalange of the third finger of his left foot, the distal phalanges of second, third and fourth fingers of his right hand and the distal phalange of first finger of his right foot (Fig. 1). In all these cases, pathological examination described a chronic granulomatous flogistic process, that involved entirely all the anatomical structures (from bone to skin), with epitelioid histiocytes, multinuclear giant cells surrounded by lymphocytes, and in some granulomas central necrosis (Fig. 2). The lesions tended to recur in other fingers of his hands and feet, and at proximal metacarpophalangeal joints of fingers amputated of distal parts; in addition, for several years a rubbery non erythematosus nor painful plaque was present, without modifications in the left perimalleolar region (Fig. 3). Because of the persistence of symptoms and the concomitant appearance of chronic chest pain with dyspnoea, the patient came to our attention with the suspect of a systemic granulomatous disease, so that sarcoidosis, mycobacteriosis and Hansen disease were taken into account. Routine blood tests were normal for age and sex, except the increase of CA-19.9, (53 U/mL, n.v. <39 U/mL). Chest X-ray showed a tenuous opacity in right supra-diaphragmatic area. This finding was not confirmed at the high resolution chest tomography (HRCT), in which parenchyma of lung was normal, while mediastinal lymph nodes were enlarged. A subsequent chest contrast CT confirmed the presence of enlarged lymph nodes tending to conglutination and with calcifications inside.

Ecg was normal, while echocardiography showed a little trans-tricuspid regurgitation and an assessed PAPs of 25 mmHg. Neurological examination was normal. Eye examination did not show significant pathologic findings. Blood gas analysis was normal for age, six minutes walking test was performed without pathological desaturation and with an appropriate distance walked in relation to age and sex; a spirometry with DL_CO_ was performed, reported as normal in relation to age, sex and haemoglobin value. Mantoux skin test and IGRA test (Elispot-TB) were negative. PET/CT scan total body was performed showing amount of radiotracer in upper lobe of right lung (SUV max 4,01), mediastinal nodes (SUV max 6,70), skeleton in several upper and lower limbs’ bones segments and metacarpophalangeal joints of his hands and feet. On the basis of these findings a bronchoscopy was executed with transbronchial needle aspiration (TBNA) of mediastinal affected lymph nodes, bronchoalveolar lavage (BAL) and bronchoaspiration for cytological and microbiological examination, including M. Tuberculosis and other mycobacterium species; pulmonary CD4/CD8 ratio was less than 3,5.

Biopsies of finger nodules and perimalleolar plaque were performed for pathologic and microbiological tests, including amplification of microbial DNA by PCR technique. The results confirmed the same granulomatous aspect of nodules previously removed, without any evidence of microbial presence.

Given the clinic and laboratory results, the patient was treated with prednisone *per os* at the dose of 0.5 mg/kg/die for one month, reducing the dose to 0.35 mg/Kg/die after one month, and finally to 0.2 mg/Kg/die in the third month. After one month of therapy, a reduction of inflammation was observed, the nodules seemed to be brighter and much less painful; whereas in the third month, we found a total healing, with scar formation. After six months from the beginning of therapy, blood tests were normal; a new PET/CT revealed the 18 F-fluorodeoxyglucose uptake in the region of mediastinum with a maximum SUV of 3.8 with a SUV lower than first PET/CT in the fingers of his hands; chest HRCT was unmodified. The patient is still in clinical, functional and radiological follow up, and currently in stable conditions.

## Discussion

Osteoarticular involvement in sarcoidosis is uncommon [1, 2, 3]. The incidence is around 3–13 % and is clinically relevant only in 2–5 % of cases, often presenting an asymptomatic course [4, 5]. A wide, clinical variability is described in symptomatic cases, from minor manifestations, sometimes self-limiting, to osteolysis and pathologic fracture with involvement of the surrounding soft tissues. The most affected sites are the small joints of hands and feet, and patients with bone involvement often have also lung disease (up to 90 % of cases) and skin involvement (in a proportion that rises to 25 %) mainly in the form of Lupus Pernio. Various forms of osteoarticular involvement of sarcoidosis are described in the literature. One of them is the dactylitis, which often appears as other rheumatological diseases, such as psoriatic arthritis, especially in the black population, with or without involvement of the overlying skin [6]. Another joint involvement is certainly acute arthritis that affects up to 40 % of patients with sarcoidosis with joint involvment, especially in the early stages of the disease; when it is in the form of acute polyarthritis associated with lymphadenopathy and erythema nodosum is called Löfgren syndrome [7]. This form is more common in women, is often symmetric, oligoarticular and hardly affects the small joints of the hands and feet, often self-limiting. The multiple cystic osteitis or Jungling syndrome shows a typical radiological pattern, with cystic rounded lesions; rarely it is periosteal reaction [8], while in more severe forms of the disease pathological fractures and osteolysis may be present [9]. Chronic arthritis is a rare event, affecting mainly black males and typically occurs in association with involvement of other organs such as lungs, skin (Lupus pernio), and eyes (chronic uveitis) [10]. This form of arthritis has various manifestations, such as non deforming arthritis with granulomatous synovitis, Jaccoud disease (deviation of metacarpal finger, metacarpophalangeal joint subluxation and deformations “swan neck” of the small joints), or Rheumatoid arthritis-like forms, with involvement of periarticular soft tissues. In these cases differential diagnosis is crucial, mainly based both on patient's clinical history, and on serology in order to search specific autoantibodies [11]. Despite the wide clinical variability of the osteoarticular sarcoidosis, all forms of the disease are widely benefited from a therapy with corticosteroids, which allow a firm remission of the pathology (similarly to what occurs in other involved organs) and a marked improvement of symptoms (pain, functional limitation and bone destruction) [12, 13]. In our case, the patient came to our attention after surgical amputation of his fingers of hands and toes affected by the disease, with a new presentation of the disease on the surgical stumps associated with mediastinal lymphadenopathy. The involvement of the interphalangeal joints in combination with pathology of soft tissue and mediastinal lymph-nodes enhances the suspicion of osteoarticular sarcoidosis, although the lungs and skin appear to be free from injury [14, 15]. Therefore, in order to assess the diagnosis of articular sarcoidosis, other rheumatologic and granulomatous diseases have been excluded, including infections (tuberculosis and others). Finally, it is very interesting to observe the total clinical remission in surgical stumps after adequate corticosteroid therapy, which represents a diagnostic confirmation *ex adiuvantibus*.

## Conclusions

This clinical case shows how sarcoidosis can appear with a wide, clinical variability [16, 17]. Osteoarticular involvement may be evident in rare forms but also aggressive to the soft tissues surrounding without substantial involvement of the lung. Currently, there are few studies addressed to appropriate therapy in these forms, although corticosteroids appear to be useful to control the disease and to prevent its rapid evolution towards forms of irreversible tissue destruction. Early identification of a joint involvement of sarcoidosis is important for an adequate diagnosis and especially for an appropriate therapy in order to prevent irreversible tissue destruction and disabling symptoms. In our case it was impossible to identify which form of osteo-articular disease the patient was affected by, and mainly which was the site primitively involved (bone? joint?), since the process *ab initio* showed to be particularly aggressive, producing a significant involvement and destruction of both tissues.

The patient responded very well to the treatment with corticosteroids, enlightening that such a drug is the treatment of choice of sarcoidosis, as well known, even after a delay in diagnosis, and even in case of particularly aggressive manifestations.

Finally, this case is to be considered, in our opinion, very unusual, both for the exclusive osteo-articular localization, and for the rapid aggressiveness, leading to destructive lesions.

## Consent

“Written informed consent was obtained from the patient for publication of this Case report and any accompanying images. A copy of the written consent is available for review by the Editor-in-Chief of this journal.”

## Abbreviations

TBNA: transbronchial needle aspiration
HRCT: high resolution chest tomography
CT: computerized tomography
PAP: pulmonary arterial pressure
IGRA: interferon gamma release assay
PET: positron emission tomography
PCR: polymerase chain reaction
DLCO: carbon monoxide diffusing capacity
BAL: bronchoalveolar lavage
